# Tandem application of endophytic fungus *Serendipita indica* and phosphorus synergistically recuperate arsenic induced stress in rice

**DOI:** 10.3389/fpls.2022.982668

**Published:** 2022-09-06

**Authors:** Shafaque Sehar, Qidong Feng, Muhammad Faheem Adil, Falak Sehar Sahito, Zakir Ibrahim, Dost Muhammad Baloch, Najeeb Ullah, Younan Ouyang, Yushuang Guo, Imran Haider Shamsi

**Affiliations:** ^1^Zhejiang Key Laboratory of Crop Germplasm Resource, Department of Agronomy, College of Agriculture and Biotechnology, Zhejiang University, Hangzhou, China; ^2^Dow International Medical College, Dow University of Health Sciences, Karachi, Pakistan; ^3^Faculty of Agriculture, Lasbela University of Agriculture, Water and Marine Sciences, Uthal, Pakistan; ^4^Faculty of Science, Universiti Brunei Darussalam, Bandar Seri Begawan, Brunei; ^5^China National Rice Research Institute (CNRRI), Fuyang, China; ^6^Guizhou Academy of Tobacco Science, Guizhou, China

**Keywords:** arsenic toxicity, endophytic fungus, phosphorous nutrition, *Oryza sativa*, antioxidant enzymes, gene expression analysis

## Abstract

In the context of eco-sustainable acquisition of food security, arsenic (As) acts as a deterring factor, which easily infiltrates our food chain *via* plant uptake. Therefore, devising climate-smart strategies becomes exigent for minimizing the imposed risks. Pertinently, *Serendipita indica* (*S. indica*) is well reputed for its post-symbiotic stress alleviatory and phyto-promotive potential. Management of phosphorus (P) is acclaimed for mitigating arsenic toxicity in plants by inhibiting the uptake of As molecules due to the competitive cationic exchange in the rhizosphere. The current study was designed to investigate the tandem effects of *S. indica* and P in combating As toxicity employing two rice genotypes, i.e., Guodao-6 (GD-6; As-sensitive genotype) and Zhongzhe You-1 (ZZY-1; As-tolerant genotype). After successful fungal colonization, alone and combined arsenic (10  μ M L^−1^) and phosphorus (50  μ M L^−1^) treatments were applied. Results displayed that the recuperating effects of combined *S. indica* and P treatment were indeed much profound than their alone treatments; however, most of the beneficial influences were harnessed by ZZY-1 in comparison with GD-6. Distinct genotypic differences were observed for antioxidant enzyme activities, which were induced slightly higher in *S. indica*-colonized ZZY-1 plants, with or without additional P, as compared to GD-6. Ultrastructure images of root and shoot exhibited ravages of As in the form of chloroplasts-, nuclei-and cell wall-damage with enlarged vacuole area, mellowed mostly by the combined treatment of *S. indica* and P in both genotypes. Gene expression of PHTs family transporters was regulated at different levels in almost all treatments across genotypes. Conclusively, the results of this study validated the promising role of *S. indica* and additional P in mitigating As stress, albeit corroborated that the extent of relevant benefit exploitation is highly genotype-dependent. Verily, unlocking the potential of nature-friendly solutions will mend the anthropogenic damage already been done to our environment.

## Introduction

Rice (*Oryza sativa* L.) stands out among the cereals as the 3rd most commonly cultivated crop across the globe with a production of 211 million metric tons in 2020, spreading across 164.19 m ha (FAOSTAT, 2021). Most concerning, the World Population Prospects report, promulgated by the Department of Economic and Social Affairs (UNDESA, 2019), presents an extensive review of the future global demographic trends/prospects, according to which the world population is expected to reach 9.8 billion in 2050 and the statistical ascension of population size is projected to persist. Unfortunately, the available arable land is actually reducing in size and rice production is under threat due to the heavy metal (HM) pollution (e.g., cadmium, arsenic, lead, etc.), brought about by human activities (Adil et al., 2020a). As a matter of fact, about 1/3rd of the world’s cultivable land has been lost due to these reasons in past 4 decades with potentially devastating consequences on the horizon as the global food demand rises exponentially (Ahmad et al., 2014; Delang, 2018). Arsenic toxicity is prevalent in rice production due to the considerable amount of water required for a production cycle (Nagajyoti et al., 2010). Arsenic is an exceptionally harmful and ubiquitous metalloid in the environment that not only has adverse effects on agricultural production, but also on human health. Arsenate (As^5+^) is the abundant form of arsenic in aerobic environments and promotes plant cell damage *via* oxygen species (ROS) signaling pathways (Zvobgo et al., 2018). Acceptable levels of arsenic set by United States Environmental Protection Agency (USEPA) in soil range from 0.39 to 39 ppm (ATSDR, 2007). Although, the concentration of soil As varies with geographical regions, the global average is about 5 mg kg^−1^; however, the concentration could reach hundreds or thousands of mg kg^−1^ in contaminated environments (Zhao et al., 2010). Soil samples collected across China’s arable soils in 2011 and 2016 revealed that the median As concentration in surface soils was 9.7 mg kg^−1^; moreover, the total arsenic in the Chinese agricultural surface soils was inventoried to be 3.7 × 10^6^ tons (Zhou et al., 2018). Due to the increasing reports of contamination of As in the soil and water and its potential downstream health risks (Shahzadi et al., 2022), it is reasonable to investigate how As toxicity could be counterbalanced. Therefore, crop scientists have been working round the clock to help find alternatives, researching on sustainable and productivity boosting aspects of agricultural crops around the world (Shah et al., 2021; Ulhassan et al., 2022).

Fortunately, there are some ways to help plants manage As stress; these ways include beneficial microorganisms’ interaction with plants and one of the great illustration is *S. indica*, an endophytic fungus with exceptional abilities to promote plant growth indices even in stressful scenarios such as arsenic exposure (Unnikumar et al., 2013). Classified under a rather new fungal family *Sebacinaceae* and new order *Sebacinales* under the phylum *Glomeromycota* (Varma et al., 1999), *S. indica*, (previously known as *Piriformospora indica*), can colonize a wide range of crop and non-crop plants, including but not limited to rice, maize, wheat and barley (Gill et al., 2016). Ever since its discovery, *S. indica* has been the cynosure of many scientific investigations because of its phenomenal potential; Singhal et al. (2017) and Bakshi et al. (2017) have thoroughly reviewed the magnificent features hidden in the fungus, such as bio-protection, growth promotion and enhancement of plants’ defense mechanisms against abiotic and biotic stresses. Mohd et al. (2017) explored the *S. indica* colonization-mediated response of *Oryza sativa* to As toxicity and revealed that host root-fungal symbiosis promoted the rescue of total biomass, chlorophyll and root damage caused by As toxicity. *S. indica* was able to achieve this by immobilizing soluble As and restrict its infiltration to the roots, with only a small amount of As gaining access to the shoots. Its colonization led to a change in the cells’ redox status by manipulation of the antioxidative framework, thereby defending the photosynthetic apparatus of the plant from As stress (Mohd et al., 2017). As reported by Ghorbani et al. (2021), *S. indica*-colonized seedlings displayed significant reductions in malondialdehyde and methylglyoxal levels by modulating AsA, glyoxalase system and GSH homeostasis. Evidence provided by Bertolazi et al. (2019) substantiate the claim of crop growth promotion and a higher performance by means of symbiotic interactions, as they witnessed similar growth responses in their investigation with the wild-type rice plants set against H + -PPase gene overexpressing transgenic plants.

Another way to cope with arsenic toxicity is through nutrient management such as P, which can specifically interact with As due to the anionic structural similarity (Zvobgo et al., 2018). Phosphorus makes about 0.2% of a plant’s dry weight and is among the three macronutrients most essential to plants’ existence, as it partakes in many key metabolic pathways, enzymatic reactions and is the chief constituent of biological molecules such as phospholipids, nucleic acids and adenosine triphosphate. It is notable that enzymes that utilize P, have the same binding mode and kinetic parameters as As^5+^, therefore plants incur toxicity when As^5+^ substitutes P in metabolic reactions (Kamiya et al., 2013). Phosphorus has been registered as an alleviant of stress specifically As stress in plants, as P and As share common transporters (Ye et al., 2017). Rice plants lack inherently evolved As transporters (Ghorbani et al., 2021), yet arsenic manages to get easily transported across the plasma lemma by PHT proteins (Ye et al., 2017). The function and characteristics of all plant PHTs, and their roles in *Arabidopsis* and rice have been discussed in depth by Młodzińska and Zboińska (2016). A research on *Salix* spp. revealed that the expression of PHT 1; 3 and PHT 1; 12 in the absence of P, was up-regulated immediately upon As exposure (Puckett et al., 2012); moreover, some PHTs have been identified in plants that show strong association with mycorrhizal symbiosis, such as PHT11 and PHT13 (Ye et al., 2017). Previous studies attribute *S. indica*-mediated promotion of plant development to phosphate transfer, as impairment in growth resulted from plant’s inoculation with its phosphate transfer (PiPT) knock-out strains (Kumar et al., 2011). According to an experiment conducted by Ngwene et al. (2016), P utilization is increased by the endophytic root symbiont *S. indica*, particularly when the source is inorganic; moreover, the resultant symbiosis could reprogram gene expression but may not necessarily compensate for phosphate limitation (Bakshi et al., 2017), therefore an additional P supply would certainly help.

To further comprehend the role of *S. indica* in plant As toxicity amelioration, it is paramount to discern first how *S. indica* utilizes P for counteracting As toxicity, and second its capability to work together with the innate genotypic potential of the crop in question. Based on the above facts, the current study was designed with the salient objectives: (i) to study the physio-biochemical responses of rice to arsenic stress under the combined treatment of *S. indica* and phosphorus, (ii) to investigate the possible mechanism involved in the combined treatment of *S. indica* with phosphorus in alleviating arsenic stress in rice, and (iii) to explore the ultrastructural configurations of root and leaf tissues and relative gene expression involved in As-stress mitigation.

## Materials and methods

### Plant material, *Serendipita indica* culture preparation and inoculation

Healthy seeds of two rice genotypes, Guodao-6 (high As accumulator) and Zhongzhe You-1 (low As accumulator), were disinfected using 3% perhydroxic acid for about 15 min, then rinsed 3 times with ddH_2_O. Thereafter, the seeds of each genotype were soaked in a labeled germination box overnight at room temperature (25°C). Afterward, the well moist seeds were placed for germination in a rice growth chamber at 29°C ± 1°C during the day (12 h) and 26°C ± 1°C at night. A hydroponic setup was arranged and 10 days old, uniform sized seedlings were transferred to half strength nutrient solution (slightly modified from Zeng et al., 2008) for 3 days followed by full strength solution for 4 days before fungal inoculation. Nutrient solution comprised of the following chemicals (L^−1^): 2.9 mM NH_4_NO_3_, 1.7 mM MgSO_4_·7H_2_O, 1.0 mM K_2_SO_4_, 1.0 mM CaCl_2_, 0.125 mM NaH_2_PO_4_.2H_2_O, 36 μM EDTAFeNa, 18 μM H_3_BO_3_, 9.1 μM MnCl_2_·4H_2_O, 0.52 μM (NH_4_)_6_Mo_7_O_24_·4H_2_O, 0.16 μM CuSO_4_·5H_2_O, and 0.15 μM ZnSO_4_·7H_2_O. *Serendipita indica* was first grown on agar Petri plates with Aspergillus medium; circular agar disks (5 mm in diameter) inclosing active spores of *S. indica* (Hill and Kafer, 2001), were placed on solidified medium and kept in absolute dark for 7 days at 30°C ± 1°C. For inoculation, *S. indica* was grown in a liquid Käfer medium from previously inoculated Petri plates; a small circular section of about 1 mm diameter of the inoculated fungus was introduced into a 500 ml glass flask and then incubated for 12 days at 30°C ± 1°C in a shaking incubator (200 rpm). Thereafter, the liquid containing active spores was filtered through sterile muslin cloth then used for plant inoculation by root soaking method (Chadha et al., 2015).

### Confirmation of *Serendipita indica* root colonization and treatment application

**Figure 1:**
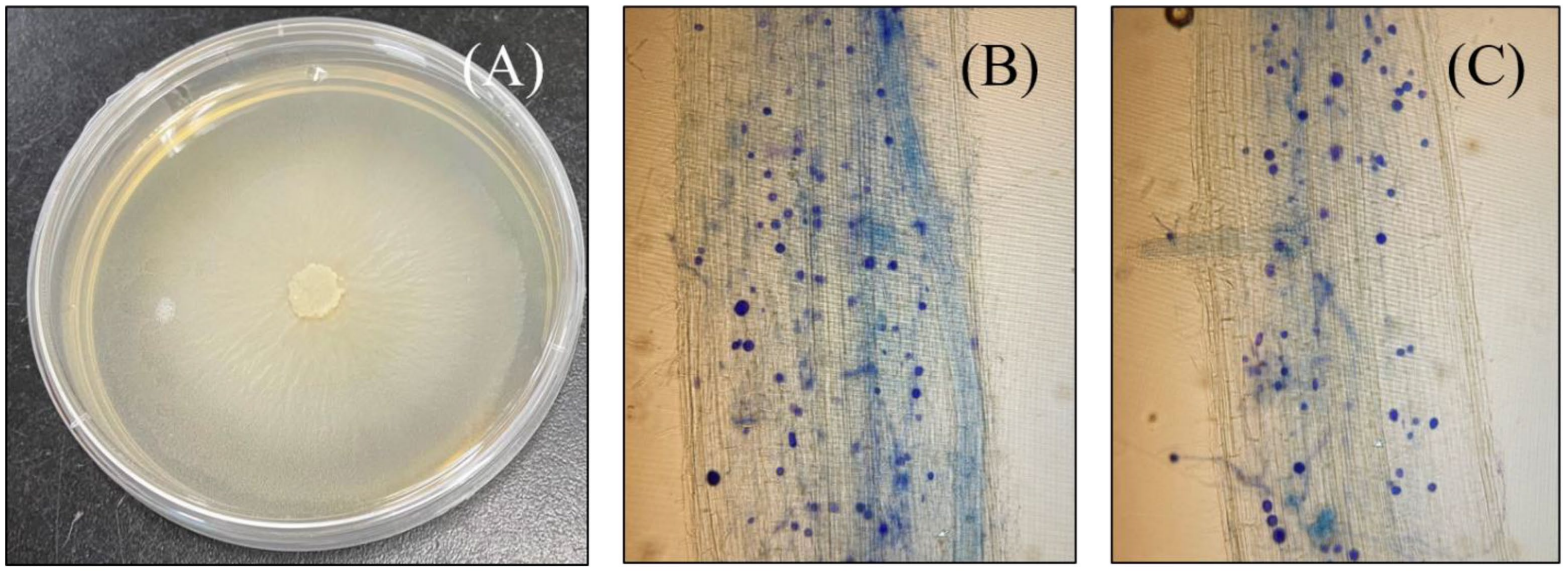
Photograph of a petri plate growing culture of *Serendipita indica* **(A)**, and microscopic images of both rice genotypes successfully colonized with *S. indica* spores; Roots of ZZY-1 genotype **(B)** and, GD-6 genotype **(C)**.

Seven-day post-inoculation, successful colonization was confirmed by microscope visualization of *S. indica* spores in the root cortical tissues of the host plant. Inoculated plant roots were first gathered and rinsed thoroughly with water before cutting them into 1 cm long pieces and then immersed in KOH solution (10%) overnight at room temperature (25°C; Chadha et al., 2015). The roots were thereafter, washed three times with ddH_2_O and then immersed again, this time with 1% HCl for about 3 min prior to trypan blue (0.05%) staining for microscopic visualization as seen in Figure 1. Followed by successful colonization, plants were kept either as positive control (*S.i*) or treated with alone 10 μM As L^−1^ (As + *S. indica*), 50 μM P L^−1^ (P + *S. indica*) and their combination (As + P + *S. indica*). A set of un-inoculated plants were also treated with either alone or combined As and P treatments (As, P, and As + P), meanwhile seedlings without any treatment were designated as control (CK). Arsenic was provided as Na_2_HAsO_4_.7H_2_O (0.0078 g L^−1^), whereas for 50 μM phosphorus, 0.25 mM NaH_2_PO_4_.2H_2_O (0.039 g L^−1^) was added (stock solutions).

### Evaluation of phenotypic and photosynthetic attributes

Measurement process and methods used were identical for both genotypes; growth indices including shoot height (SH) and root length (RL), fresh (FW) and dry weights (DW) were measured upon harvest (15-day post-treatment) taking two seedlings from each of the three biological replicates. Dried mass was measured after drying the roots and shoots separately in labeled paper bags at 75°C ± 2°C for 72 h using a hot air oven. Measurements of net photosynthetic capability (A_max_), stomatal conductance (*Gs*), transpiration rate (*Tr*), and intercellular CO_2_ concentration (*Ci*), were performed on second fully expanded leaves 24 h before harvest at day 14 post-treatment, using an infrared analyzer (LI-6400 System, Li-COR Company, United States). All measurements were performed between 9 am and 12 pm, with relative humidity of 50%–70%, CO_2_ concentration of 400 μmol mol^−1^, air temperature of 25°C to 28°C and photosynthetic photon flux density of 1,000 μmol m^−2^ s^−1^.

### Electron microscopic imaging

Ultrastructural study was performed on root and shoot of both genotypes, the samples were examined using only a tiny portion of the plant tissues, about 1 mm^2^ sections of fresh root tips and fully expanded uppermost leaves of the plants. The samples were fixed in a 1:1 mixture of 2.5% glutardialdehyde and 0.1 M phosphate buffer (PBS; pH 7.0) for 6 h and post-fixed in a 1:1 mixture of 2% OsSO_4_ and 0.1 M PBS (pH 7.0) for 2 h. A series of graded ethanol (75%, 80%, 90%, and 95%) was used to dehydrate the samples with a subsequent pure acetone wash (Mapodzeke et al., 2021). Later, the samples were infiltrated with successive combinations of Spur resin and acetone, followed by embedding in Spur resin overnight. The samples were then polymerized at 70°C for 9 h. Ultrathin sections of the samples were made and stained in uranyl acetate (C_4_H_6_O_6_U), and later by lead citrate (C_12_H_12_O_14_Pb_2_). Shoot and root ultra-structures were examined and photographed under a Hitachi H7650 transmission electron microscope.

### Determination of elemental concentration and translocation factor

Dried root and shoot samples of the two genotypes were collected (0.1 g) and placed in digestion glass tubes with high heat resistance according to their labels, then 3 mL of nitric acid (HNO_3_) were added to each sample tube and processed in a microwave (Mars 6, CEM Technologies, United States) at 120°C for 1 h 30 min. Macro-, meso- and micro-elements were then detected using an inductively coupled plasma–optical emission spectrometer (ICP-OES; Optima 8000DV; Perkin Elmer). The translocation factor (%TF) was calculated by the ratio of As total accumulation in the shoot with As total accumulation in the root as presented by Zvobgo et al. (2018).

### Antioxidant enzyme activity and estimation of lipid peroxidation

About 0.2 g of leaf tissue was homogenized with 4 mL cold 5 mM PBS (pH 7.8) and centrifuged (Eppendorf 5810R; Hamburg, Germany) at 4°C for 15 min at 12,000 rpm. The activities of SOD (EC 1.15.1.1), CAT (EC 1.11.1.6), and POD (EC 1.11.1.7) were assayed according to Wu et al. (2003). By measuring the MDA content (Murphy et al., 2014), lipid peroxidation levels were calculated; the reaction solution comprised of 5% trichloroethanoic acid solution, 2.5 g of thiobarbituric acid and enzyme extract. The final mixture was heated for 15 min at 95°C, followed by immersion in an ice water bath to halt the reaction and subsequently centrifuged for 10 min at 4,800 rpm. Lastly, the supernatant was read spectrophotometrically (BMG Labtech—SPECTROstar® Nano; Offenburg, Germany) at 532 nm (Zhang et al., 2005). Quantification of H_2_O_2_ in crude extracts was performed using the colorimetric H_2_O_2_ assay kit (A064-1), as per the manufacturer’s protocol (Nanjing Jiancheng Bioengineering Institute NJBI). Quantification of O_2_^−^ was performed using crude extracts following the method of Jiang and Zhang (2001). Frozen root/shoot tissues (0.1 g) were homogenized with 1 mL of 65 mM PBS (pH 7.8) and centrifuged for 10 min at 5,000× *g*. The incubation solution contained 65 mM PBS (pH 7.8; 0.9 mL), 10 mM hydroxylamine hydrochloride (0.1 mL), and supernatant (1 mL). Incubation at 25°C for 20 min ensued both before and after the addition of 17 mM sulfanilamide and 7 mM α-naphthylamine. Ethyl ether (4 mL) was added followed by centrifugation at 1,500× *g* for 5 min. The upper organic layer was removed and absorbance in the lower aqueous layer was read (at 530 nm). In order to calculate the production rate of O_2_^−^ from the chemical reaction of O_2_^−^ and hydroxylamine, a standard curve with NO_2_^−^ was used.

### RNA extraction, synthesis of cDNA, and qRT-PCR assay

Total RNA was extracted from 100 mg of root and shoot samples from each treatment employing TaKaRa MiniBEST Universal RNA Extraction Kit (catalog # 9767) as per the manufacturer’s instructions. Titertek-Berthold nanospectrometer (Pforzheim, Germany) was used for RNA concentration check, whereas for quality assessment, the samples were electrophoresed in 1% agarose gel running with 1X TAE buffer. To obtain cDNA, TaKaRa Prime script RT reagent Kit (catalog # RR037A) was used. The cDNA samples were assayed by quantitative real time PCR (qRT-PCR) in the Roche Light Cycler® 480 Instrument II Real Time PCR System using the TB Green® Premix Ex Taq™ II Clontech (catalog # RR820A; Takara). To calculate threshold cycle values, software provided with the qRT-PCR system was used; consequently, the quantification of mRNA levels was performed employing Schmittgen and Livak (2008) method. Ten primers were designed including the reference gene for *Oryza sativa* with genes of interest and are listed in Table 1. The genes were blasted from NCBI primer blast and designed choosing the most promising output among which phosphate transporters from PHTs families were chosen for expression across treatments. Other genes related to antioxidant activities were also selected and designed for expression to check correlation with antioxidant and ROS enzyme assays, performed through enzyme extraction and different reagent solutions.

**Table 1:** List of *Oryza sativa* specific primer sequences used in this study.

Gene name	Primer pair sequence (5–3`)	Length (bp)	Product length
*OsPHT4-1F*	ATCGAAAGGGAAGTCGCTGG	20	
*OsPHT4-1R*	ACCATCTCAACTCCGGACGA	20	109
*OsFe-SOD1-1F*	GCCAGAAGGTGGTGGGTCA	19	
*OsFe-SOD1-1R*	AGCCAGACCCCAAAAGTGATA	21	122
*OsMn-SOD1-1F*	CCGCACGCTGGCCTC	15	
*OsMn-SOD1-1R*	CGACGGTCGTCACACCC	17	147
*OsPHT1-1F*	GTGATGCTGCAAGCTCTTCG	20	
*OsPHT1-1R*	CCAGGTCGAGAAGTACGCAA	20	107
*OsHMA2-F*	ATTACACACCTGCGGTCGTT	20	
*OsHMA2-R*	GGGTGTGGACAGTACCAGTG	20	146
*OsHMA3-F*	GCAAGTCAAGCCACCCAATG	20	
*OsHMA3-R*	CCCACATTTTCCGGGTTTGG	20	86
*OsAPX-F*	CTAGGGCCAGTGTGAACCAG	20	
*OsAPX-R*	GCAGCATTGCAGTTGAGCAT	20	127
*OsCAT-F*	CTCCGTGGCATCTGGATCTC	20	
*OsCAT-R*	TTCCTCCTGGCCGATCTACA	20	108
*OsCu-ZnSOD-F*	CAGATTCCTCTGAGTGGCCC	20	
*OsCu-ZnSOD-R*	AACAACACCGCATGCAAGTC	20	141
*OsActin-1F*	AGGCCCCTTTGAACCCAAAA	20	
*OsActin-1R*	ATAGCGACGTACATGGCAGG	20	106

### Statistical analysis

A completely randomized experiment was designed with 3 biological replicates for each treatment administrated and the statistical analysis (one-way ANOVA) was performed by SPSS-10 statistical software (SPSS Inc., Chicago, IL, United States) using Duncan’s Multiple Range test (DMRT) at *p* < 0.05 (Goodall, 1991). OriginPro 2019 (Origin Lab Corporation, Wellesley Hills, Wellesley, MA, United States) was employed for PCA analysis and graphs, whereas the tables were made in Microsoft Excel 2019.

## Results

### Growth responses and photosynthetic indices

The phenotypic differences across the two rice genotypes are presented in Table 2. Growth attributes of both genotypes, i.e., fresh (FW) and dry biomass (DW) or length of roots (RL)/height of shoots (SH), were severely affected by arsenic toxicity. It is interesting to note that symbiosis of *S. indica*, in the absence of any other treatment, had a burgeoning effect on the growth of plants, although not statistically significant from their respective controls in some cases, with 15.6% and 18% increase in SFW, 16.8% and 13.7% in RFW, 16% and 14.8% in RDW, as well as 3.8% and 5.9% in RL, for ZZY-1 and GD-6 genotype, respectively; whereas, a significant difference in SDW (30.7%) was observed with *S. indica* colonization for ZZY-1, and in SH (18% and 14.6%) for both ZZY-1 and GD-6, respectively, as compared to their control plants. The combined treatment of P and *S. indica* also had a positive impact on all the growth indices except for SFW in As-stressed ZZY-1. When the ameliorative treatments were compared for their efficacy, *S.i* + As displayed 41.4% and 2.34% improvement in RL against As alone treatment, As + P showed 27% and 17.5%, whereas *S.i* + As + P had 27% and 33.4% better root growth in ZZY-1 and GD-6, respectively. Moreover, in terms of SL, ZZY-1, and GD-6 indicated a 21.9% and 17.7%, 3.8% and 3.5%, along with 0.52% and 30.3% recovery under *S.i* + As, As + P, and *S.i* + As + P treatments, respectively. A noteworthy 2.3- and 2.7-fold improvement in RDW was observed for ZZY-1 under combined *S. indica* treatments with As and As + P than alone As treatment. Moreover, GD-6 plants benefited the most from *S.i* + As + P with 66.5% lesser reduction in comparison with their alone As treated counterparts. While recording the data for shoot dry weight, it was observed that *S.i* + As brought forth a better shoot dry weight than As + P and *S.i* + As + P application (4.7- and 3.4-fold, respectively), when compared to As treated ZZY-1 plants; however, *S.i* + As + P showed a 2.5- and 2-fold higher SDW preservation compared to *S.i* + As and As + P, respectively. Against arsenic treated plants, root fresh weights were restored significantly under *S.i* + As + P application than *S.i* + As and As + P, while an opposing trend was seen in SFW values, where both *S.i* + As and As + P treatments presented higher FW (43% and 24.7% for ZZY-1, 97% and 99% for GD-6) against 2.35% and 2% under *S.i* + As + P, respectively.

**Table 2:** Growth indices of rice plants differing in arsenic accruing tendency following alone and combined interactions of *Serendipita indica* symbiosis and phosphorus against arsenic stress.

Genotype	Treatment	Shoot FW (g)	Root FW (g)	Shoot DW (g)	Root DW (g)	Shoot length (cm)	Root length (cm)
ZZY-1	CK	6.04 ± 0.71 ab	1.25 ± 0.08 a	0.673 ± 0.022 b	0.119 ± 0.03 ab	80.5 ± 4.2 b	21.3 ± 0.45 a
	As	1.24 ± 0.32 d	0.409 ± 0.09 b	0.225 ± 0.015 d	0.03 ± 0.005 d	51.9 ± 3.1 c	11.8 ± 1.7 c
	*S.i*	6.99 ± 0.95 a	1.46 ± 0.21 a	0.78 ± 0.031 a	0.138 ± 0.01 a	95 ± 2.5 a	22.1 ± 2.3 a
	P	5.58 ± 0.84 b	1.15 ± 0.34 a	0.732 ± 0.024 ab	0.118 ± 0.07 b	77.2 ± 3.8 b	19.3 ± 1.5 ab
	*S.i* + As	1.78 ± 0.23 d	0.43 ± 0.07 b	0.51 ± 0.011 c	0.097 ± 0.02 bc	63.3 ± 4.23 c	16.7 ± 0.9 b
	As + P	1.55 ± 0.11 d	0.41 ± 0.01 b	0.29 ± 0.016 d	0.041 ± 0.006 d	53.9 ± 1.9 c	15.1 ± 1.25 bc
	*S.i* + P	4.92 ± 0.45 bc	1.69 ± 0.25 a	0.685 ± 0.042 b	0.137 ± 0.04 a	83.9 ± 4.9 ab	20.7 ± 2.1 a
	*S.i +* As+ P	4.17 ± 0.21 c	1.02 ± 0.41 ab	0.275 ± 0.001 d	0.11 ± 0.055 bc	52.2 ± 5.62 c	15 ± 2.4 bc
GD-6	CK	4.19 ± 0.52 a	1.23 ± 0.25 b	0.58 ± 0.065 abc	0.117 ± 0.04 b	62 ± 2.8 b	16.3 ± 1.7 a
	As	1.12 ± 0.34 c	0.592 ± 0.06 c	0.32 ± 0.028 e	0.056 ± 0.01 d	47.6 ± 2.3 d	12.8 ± 0.95 c
	*S.i*	4.94 ± 0.61 a	1.42 ± 0.34 ab	0.671 ± 0.11 ab	0.134 ± 0.012 ab	69.4 ± 1.78 a	17.3 ± 1.1 a
	P	3.97 ± 0.74 ab	1 ± 0.08 bc	0.54 ± 0.02 c	0.107 ± 0.06 bc	55.8 ± 3.5 bc	17 ± 0.89 ab
	*Si* + As	2.2 ± 0.15 bc	0.735 ± 0.02 c	0.363 ± 0.041 de	0.069 ± 0.003 cd	56 ± 1.2 bc	13.1 ± 1.02 c
	As + P	2.22 ± 0.46 bc	0.669 ± 0.07 c	0.372 ± 0.023 de	0.059 ± 0.017 d	49.2 ± 2.7 cd	15 ± 0.11 b
	*S.i* + P	5.1 ± 0.82 a	1.14 ± 0.09 b	0.756 ± 0.09 a	0.167 ± 0.028 a	68.9 ± 1.14 a	16.2 ± 1.5 a
	*S.i* + As+ P	3.41 ± 0.69 ab	1.75 ± 0.38 a	0.472 ± 0.02 cde	0.094 ± 0.003 c	60.5 ± 1.69 b	15.9 ± 1.13 a

All values are means of three replications (±SE). Different letters represent significant difference among the treatments within each genotype at *p* ≤ 0.05 probability level by applying Duncan’s multiple range test. CK, control; S.i, *Serendipita indica*.

**Figure 2:**
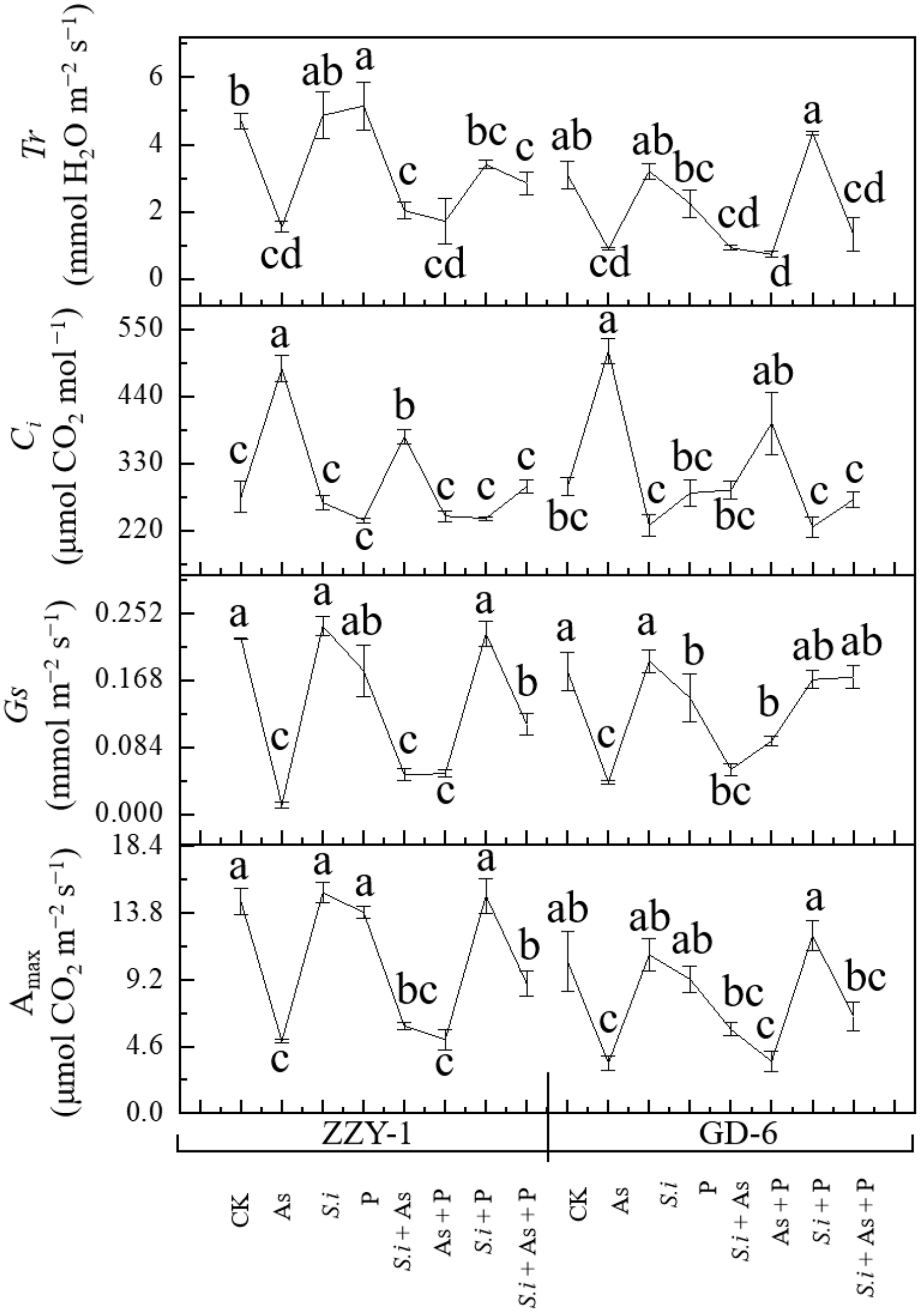
Photosynthetic attributes of two rice genotypes differing in As accumulation tendency following alone and combined interactions of *Serendipita indica* symbiosis and phosphorus against As stress. Error bars represent standard errors (n=3). Different letters represent significant difference (*p* ≤ 0.05) among the treatments within each genotype.

The effects of all treatments across rice genotypes on A_max_, *Gs*, *Ci*, and *Tr* under As stress are illustrated in Figure 2. Arsenic stress, as expected significantly reduced net A_max_ (66% and 58%), stomatal conductance (93% and 77%), and transpiration rate (66% and 71%) compared to control plants in ZZY-1 and GD genotypes, respectively. Furthermore, the combined treatment of *S. indica* and phosphorus under As stress showed much improvement in net photosynthetic rate (41%) when compared to *S.i* + As and As + P, in ZZY-1 genotype. However, GD-6 plants took advantage of *S. indica* alone (*S.i* + As; 22.7%) and in combination with phosphorus (*S.i* + As + P; 37.8% improvement), when compared with their As treated counterparts. The results indicated better performance of GD-6 under *S.i* + As + P (values closer to control) in terms of *Gs* in contrast to ZZY-1 genotype (48% revival). Although, As stress caused an elevation in intercellular CO_2_ concentrations, ZZY-1 experienced a recuperative effect under As + P (116%), *S.i* + As + P (91%), and *S.i* + As (49%); while for GD-6 plants, *S.i* + As + P tended to ameliorate the most (109%). It was interesting to note that transpiration rate was highly improved under *S.i* + As + P, and *S.i* + As treatment (41.3% and 15.4% higher compared to As treated plants, respectively) for ZZY-1 genotype, whereas for GD-6, arsenic stressed plants benefited mostly under *S. indica* symbiosis combined with phosphorus (i.e., *S.i* + As + P; 20% improvement).

### Effects of the treatments on root and leaf cell ultrastructural fingerprints

**Figure 3:**
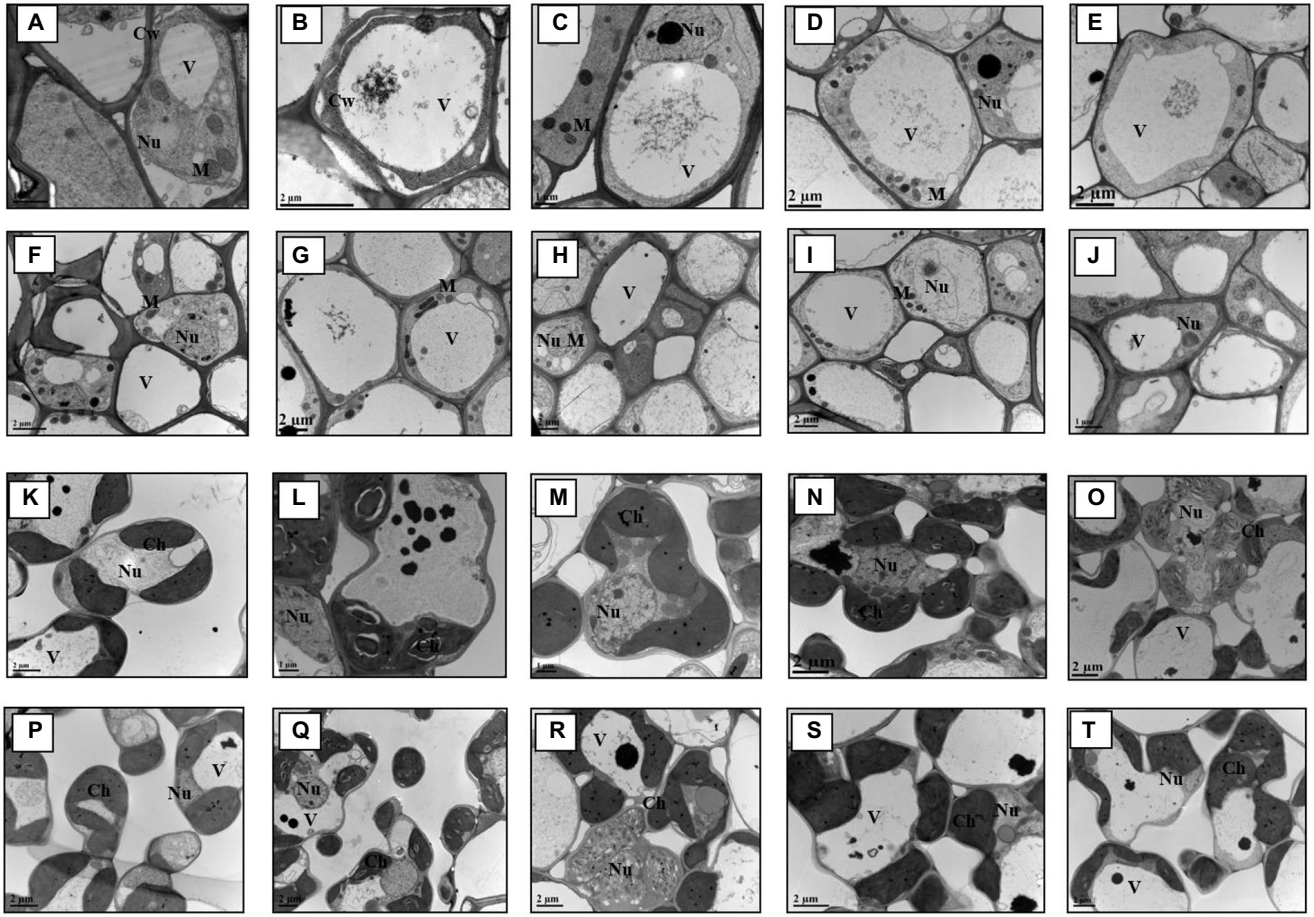
Transmission electron micrographs of root and leaf cells. Key: **(A–J)** = Root; GD-6: A = Control; B = As; C= *Serendipita indica +* As; D = *S. indica* + As + Phosphorus; E = As + Phosphorus; ZZY-1: F=Control; G = As; H=*S. indica +* As; I = D=*S. indica* + As + Phosphorus; J = As + Phosphorus. **(K–T)** = Leaves; GD-6: K=Control; L = As; M = *S. indica +* As; N=*S. indica* + As + Phosphorus; O = As + Phosphorus. ZZY-1: P=Control; Q = As; R = *S. indica +* As; S=*S. indica* + As + Phosphorus; T = As + Phosphorus.

The ultrastructure images of the root and shoot tissues present noticeable differences on cellular structure level among the treatments and across genotypes (Figure 3). It is apparent that most cellular organelles, such as mitochondria, chloroplast etc. in the control plant tissues, both in root and shoot, present normal morphology. However, other treatments in combination with As showed negative impact on plant cell organelles structures with the most damaging effect being in the cells of GD-6 genotype. Furthermore, the combined treatment of *S. indica* and P in addition to As, in particular showed greater phenotypic difference in GD-6 genotype (As-sensitive) and less damaging effect in ZZY-1. Arsenic deposition in vacuole is also visible in most of the ultrastructure images across treatments with increased vacuole area and in some, complete cell destruction.

### Concentration of macro, meso, and micronutrients

**Figure 4:**
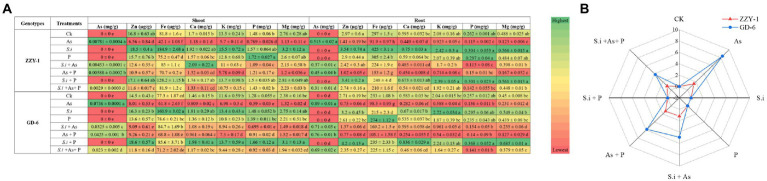
Macro-, meso-, and micro-element concentration **(A)**; green in color scale displays higher values and red shows lower values. Radar chart representing % As translocation factor **(B)**. Different letters represent significant difference (*p* ≤ 0.05) among the treatments within each genotype.

Mineral concentrations of root and shoot As, zinc (Zn), iron (Fe), calcium (Ca), potassium (K), phosphorus (P) and magnesium (Mg) were measured and the effects of all treatments applied to both rice genotypes are shown in Figure 4A, whereas percentage of arsenic translocation is also depicted alongside (Figure 4B). In shoots of ZZY-1, the ameliorative treatments, i.e., *S.i* + As, As + P and *S.i* + As + P, instigated a reduction of 42%, 24.7%, and 62.8% of arsenic concentration as compared to As alone stress, whereas for GD-6, 56.5%, 42.2%, and 68.7% decrease was observed, respectively. Roots of ZZY-1, displayed a decline of 28%, 12.6%, and 39.8% in comparison with its respective As treated counterparts, while GD-6 roots also exhibited an attenuative trend to a lesser extent (20.2%, 14.6%, and 22.4% under *S.i* + As, As + P and *S.i* + As + P, respectively). Regardless of the genotype, symbiosis of *S. indica* alone and in combination with phosphorus, tended to increase the concentration of Zn, Fe, Ca, Mg, and K, predominantly in shoots than the roots. The concentration of Zn in ZZY-1 and GD-6 shoots reduced significantly under As stress (30.8% and 18.6%, respectively), however *S.i* + As + P treatment rescued both genotypes (49.4% and 58% improvement, respectively) when compared to their respective As alone treated counterparts, while *S.i* + As and As + P proved to be more promising for ZZY-1 (58.5% and 42.6% recovery, respectively) than GD-6 (16.5% and 19.2%, respectively). In case of roots, although not much assistance was provided by the additional supplementation of phosphorus against As, the symbiosis of *S. indica* (*S.i* + As + P) remarkably improved the Zn contents in ZZY-1 and GD-6 roots (85.2% and 81.6%, respectively), followed by *S.i* + As with 64.7% and 31.7% recovery for the former and later genotype, respectively.

The calculations regarding percent translocation factor of arsenic revealed a higher reduction in GD-6 than ZZY-1; lowest As TF was observed under *S.i* + As + P (38.3% reduction) followed by *S.i* + As (19.3%) and As + P (13.8%) in ZZY-1 plants, whereas for GD-6, *S.i* + As + P was highly effective (59.7% reduction) compared to *S.i* + As (45.5%) and As + P (33.3%). Iron concentration reduced drastically under As stress for both genotypes irrespective of the tissue analyzed. It is, rather impressive to observe that treatments with *S. indica* brought about much improvement, and in few cases, higher than control Fe concentration values when compared to phosphorus amended treatments. Meanwhile, the concentration of phosphorus was higher under the treatments with augmented phosphorus as well as under *S. indica* inoculated plants and the beneficial impact was much pronounced in GD-6 shoots than ZZY-1 with 15.6% and 30% increment under *S.i* and *S.i* + P, respectively. Roots accumulated more phosphorus under alone and combined *S.i* and P treatments in both genotypes, whereas the inhibitory effect of As against phosphorus was counteracted prominently by As + P (23.8%) and *S.i* + As + P (18% improvement) in ZZY-1 compared to its As treated counterparts when set against GD-6 roots (with only 14.9% and 4.5% retrieval, respectively). A significant elevation in the concentration of shoot Ca was displayed by ZZY-1 plants under *S.i* (1.92%) and *S.i* + As (22.8% increase), whereas for GD-6, it was observed for *S.i* (23.9%) and *S.i* + P treated plants (35.3% increase). When arsenic treatment was compared with control, a 30.4% and 44.6% reduction was recorded for ZZY-1 and GD-6 shoots, while roots exhibited 24.5% and 51.6% decline in Ca content, respectively. Among the ameliorative treatments, *S.i* + As + P offered a 28% and 55.4% as well as 62.3% and 59% improved Ca accumulation in ZZY-1 and GD-6 shoots and roots, respectively. Unfortunately, in terms of K concentration restoration, As + P failed to deliver, not only in shoots but also roots of both genotypes. Concentration of Mg saw a sharp decline under As stress, where a 59.2% and 44.7% drop was observed in shoots and 74.8% and 48.6% in roots of ZZY-1 and GD-6, respectively. Shoots of ZZY-1 experienced much improvement in Mg content under *S.i* + As (62.9%) and *S.i* + As + P (67.5%) than As + P (only 4.5%), albeit GD-6 shoots were able to benefit mainly from *S.i* + As + P (58.2% recovery) as *S.i* + As and As + P did not yield much improvement (16.4% and 19%, respectively); almost a similar pattern was observed for root Mg concentration in both genotypes.

### Construing the activities of reactive oxygen species scavenging enzymes

**Figure 5:**
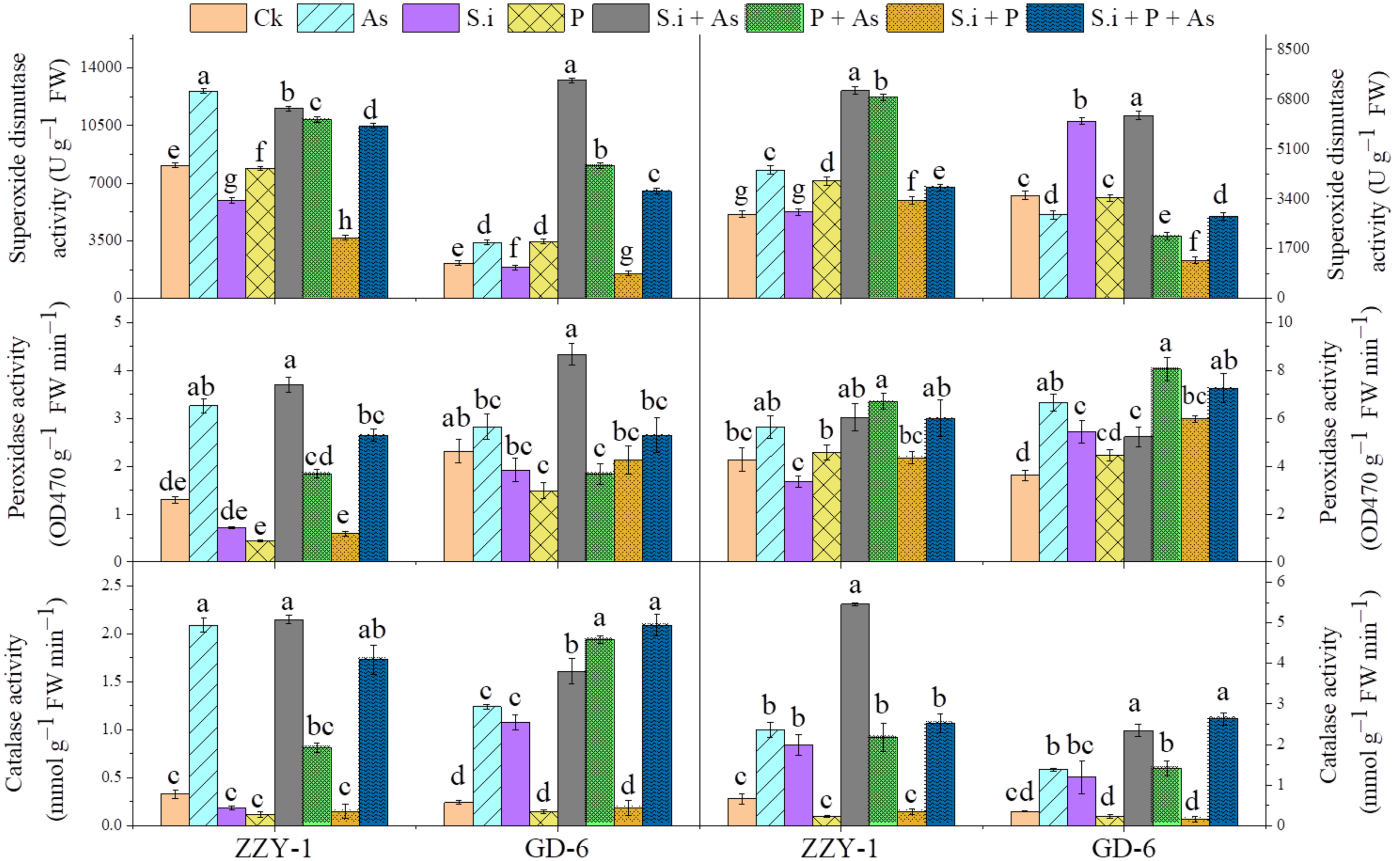
Levels of ROS scavenging enzymes in two rice genotypes differing in As accumulation tendency following alone and combined interactions of *Serendipita indica* symbiosis and phosphorus application against As stress. Right panel represent root and left shows analysis of antioxidant enzymic activities in leaves. Vertical bars represent the means of three independent replicates (±SE). Different letters represent significant difference (*p* ≤ 0.05) among the treatments within each genotype.

An increase of 5.3- and 2.6-fold was observed for catalase enzyme activity under As stress in ZZY-1 shoot and root, respectively (Figure 5); ameliorative treatments also brought forth significantly high levels of CAT activity, i.e., 5.5-, 1.45-, and 4.2-fold increase in shoots and 7.2-, 2.3-, and 2.8-fold in roots under *S.i* + As, As + P and *S.i* + As + P, respectively. Contrariwise, the *S.i* and P alone and combined treatments in the absence of As produced a downregulatory response in the activity of aforementioned enzyme, which was true for the ZZY-1 shoot but not completely for its roots, as *S. indica* symbiosis indeed raised the enzymic levels of CAT up to 2-fold; additionally, a 3.48- and 2.4-fold spike was also witnessed for GD-6 shoots and roots, respectively. Arsenic treatment triggered the activity of guaiacol peroxidase significantly in shoots and roots of both genotypes; moreover, the alleviatory treatments, predominantly *S.i* + As, also played their part in enhancing the POD levels in shoots (184% and 87.4% for ZZY-1 and GD-6, respectively), although the combined treatment (*S.i* + As + P) showed a comparatively lower increment both in shoots and roots of ZZY-1. Additionally, roots of both genotypes exhibited an increased level of POD activity under As + P (56% and 122.4% for ZZY-1 and GD-6, respectively). Compared to control, regardless of the treatment and plant tissue analyzed, the enzyme activity of superoxide dismutase increased with highest values obtained under *S.i* + As (42.3% and 147% in ZZY-1; 513% and 76.7% in GD-6, shoot and root, respectively) among the mitigatory treatments.

### Reactive oxygen species and malondialdehyde levels

**Figure 6:**
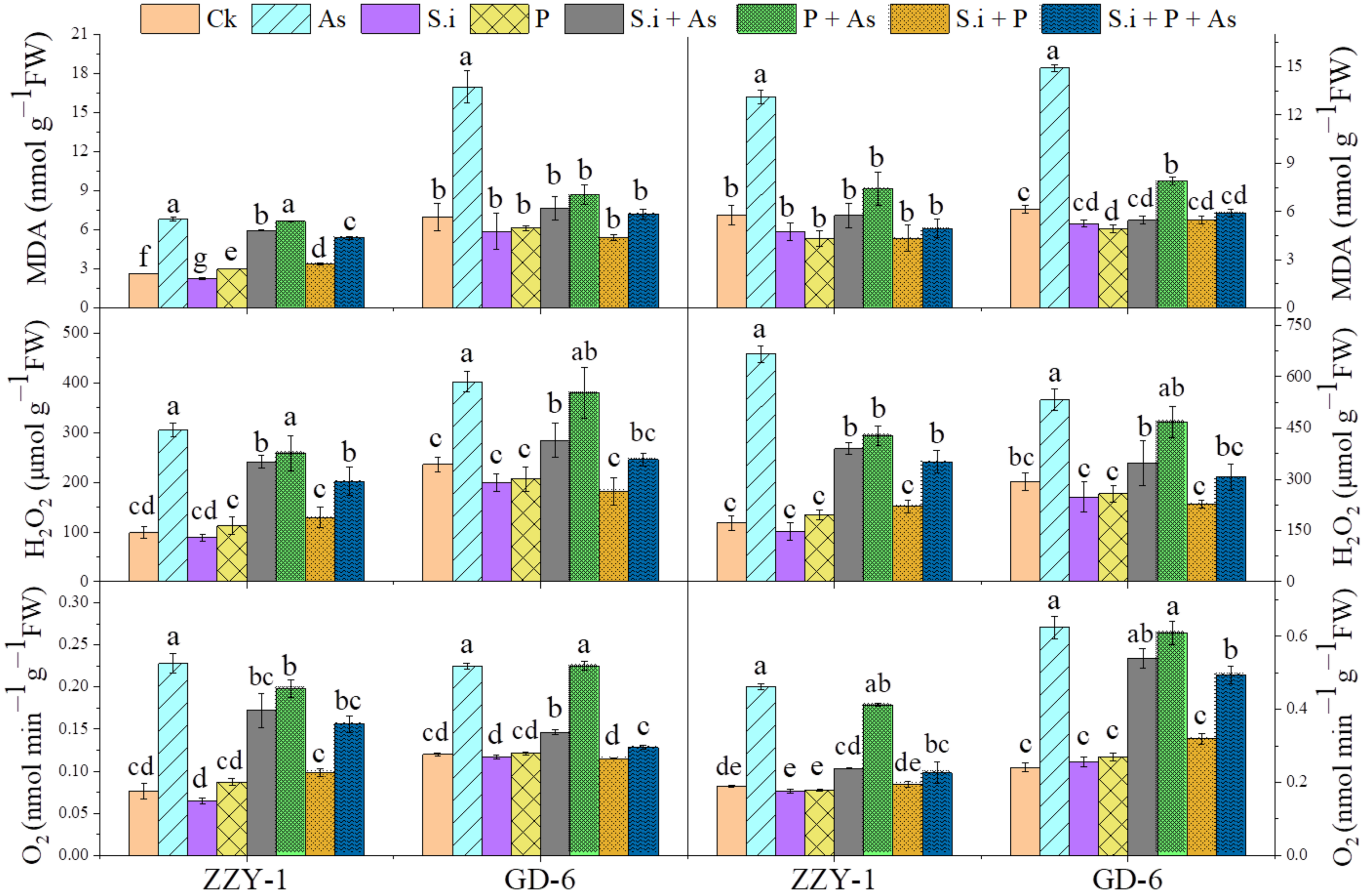
Arsenic stress-mediated oxidative burst amelioration through alone and combined interactions of *Serendipita indica* symbiosis and phosphorus application in two rice genotypes differing in As accumulation tendency. Right panel represent root and left shows analysis of ROS levels in leaves. Vertical bars represent the means of three independent replicates (±SE). Different letters represent significant difference (*p* ≤ 0.05) among the treatments within each genotype.

**Figure 7:**
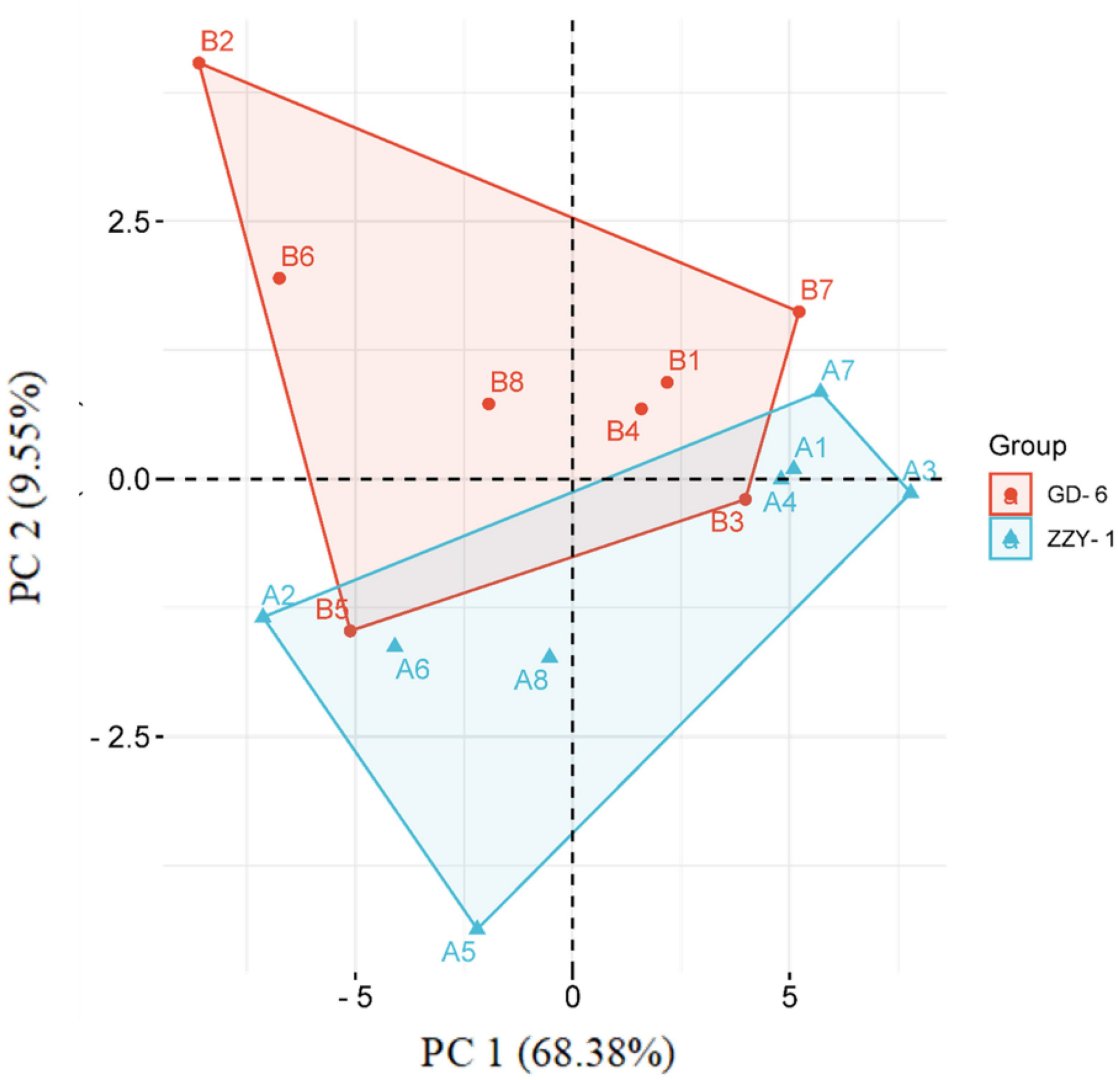
A PCA-contingent biplot of measured physio-biochemical parameters under alone and combined *Serendipita indica* symbiosis and phosphorus application. Key: A = ZZY-1 genotype; B = GD-6; numbers 1 to 8 correspond with CK, As, *S.i*, P, *S.i* + As, P + As, *S.i* + P, and *S.i* + P + As, respectively. Sh, Shoot/leaves; R, Root.

As presented in Figure 6, all the treatments except for As + P treatment assisted in reducing the lipid peroxidation caused by As toxicity in both above and below ground parts of ZZY-1 and GD-6 genotype. Of note, a 31%, 22.3%, and 49.6% drop in hydrogen peroxide content was observed for ZZY-1 shoots, meanwhile for GD-6, a 71%, 13.2%, and 93.9% decrease was recorded under *S.i* + As, As + P and *S.i* + As + P, respectively. In regard to roots, the abovementioned ameliorating treatments rendered a higher percentage of reduction in H_2_O_2_ content for both genotypes when compared to their As-stressed counterparts. Furthermore, across the genotypes both in shoot and root, a decline in the superoxide (O_2_^·−^) was detected (ZZY-1: 36.8%, 19.7%, and 47.4% in shoots, 22%, 3.9%, and 34% in roots; GD-6: 23.5%, 85.3%, and 97% in shoots, 33.3%, 69.4%, and 50% in roots) when plants under *S.i* + As, As + P and *S.i* + As + P were compared with their respective As-treatments, respectively. Reasonably, there was a strong correlation among A2, A5, A6, B2, B5, and B6 when analyzed through principal component analysis (Figure 7), then among A1, A3, A4, A7, B1, B3, B4, and B7, while A8 and B8 had inter-correlation with the two aforementioned groups.

### Relative gene expression pattern

**Figure 8:**
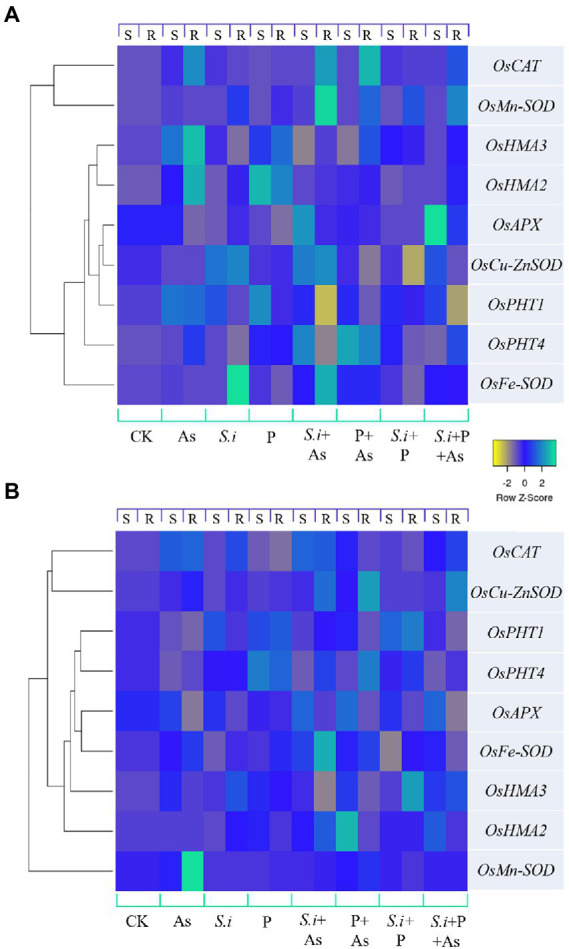
Expression levels of certain antioxidant enzymes, phosphate and heavy metal transporter genes under alone and combined *Serendipita indica* symbiosis and phosphorus application in two rice genotypes displaying different As accumulation tendencies. Color scale shows Z values, where yellow depicts downregulatory and light blue shows upregulatory trend. **(A)** GD-6 genotype and **(B)** ZZY-1; S, Shoot; R, Root.

The heat map of genes expressed differentially is presented in Figure 8. Different colors indicate different gene expressions, with light blue being the most up-regulated genes and yellow, the ones least expressed or downregulated. For GD-6 (Figure 8A) and ZZY-1 (Figure 8B) genotypes, relative expression of genes was determined based on the calculated value from the reference gene with up-regulated genes being ≥1 and downregulated genes being <1, with control plants having relative gene expression value equal to 1. A downregulatory trend in the phosphate transporters was observed with the onset of As stress in ZZY-1 genotype shoot as well as roots, in contrast with GD-6 root and shoot tissues where more than 2-fold increase was witnessed for *OsPHT1* gene expression levels. Alone and combined application of *S. indica* and phosphorus enhanced the expression of the phosphate transporters as well. In GD-6 shoots, a higher expression of *OsPHT4* was recorded for both *S.i* + As and P + As treatments (4.1- and 4.8-fold increase), while in roots it was mostly in the later treatment (4.2-fold). Regarding ZZY-1 plants, the elevation in the expression of phosphate transporters was mainly observed in roots under treatments with phosphorus supplementation with (2.7-fold increment) or without As treatment (2.3-fold increase). Considering the antioxidative capacity, there was a surge of relative expression in *OsFe-SOD*, *OsMn-SOD*, *OsCu-ZnSOD*, *OsAPX*, and *OsCAT* in both roots and shoots of ZZY-1 under *S.i* + As, P + As and *S.i* + As + P treatments. Whereas for GD-6, As combined with *S.i* or with *S.i* + P yielded the most augmentation in both the tested tissues, particularly the expression levels of *OsMn-SOD* and *OsCAT* with 15.5- and 11.8-fold increase in roots under *S.i* + As, respectively. Note-worthily, a fold change of 8.2 and 3.6 was exhibited by the roots of ZZY-1 under As stress, while the shoots presented a 1.7- and 3.4-fold increase in the expressions of *OsMn-SOD* and *OsCAT* genes as opposed to the control plants, respectively. Moreover, the expression of *OsHMA2* and *OsHMA3* was pronounced in As + P (4.5- and 1.97-fold increase, respectively) and *S.i* + As + P treatments (2.95- and 2-fold, respectively) in leaves, albeit roots displayed a 2.8-fold increase under *S.i* + As, and 1.5- and 3.2-fold as well as 1.2- and 2.3-fold relative increase of *OsHMA2* and *OsHMA3* gene expression under *S.i* + P and *S.i* + As + P, respectively. Arsenic treatment impacted the *OsHMA2* and *OsHMA3* genes in almost all the treatments in GD-6 roots and leaves. Of note, a 4.7- and 3.8-fold increase under As stress was observed in roots along with 3.9- and 2.7-fold increase under phosphorus alone treatment; a similar impact extended toward leaves as well with 2- and 2.9-fold elevation in expression of *OsHMA2* and *OsHMA3* under As stress, while 4.9- and 2.1-fold increase was seen under phosphorus fortification.

## Discussion

Most biotechnologists display a great deal of interest in the use of genetically engineered plants with superior yields and improved traits in agriculture considering it safe. However, the public abhorrence of transgenic plants and the fragmentary knowledge regarding post-consumption hazards calls for substitutes that are eco-friendly and, at the same time, cost-effective (Faiz et al., 2022). Interestingly, genotype-dependent alterations became apparent with the onset of As stress as a downregulatory trend in relative expression of phosphate transporter genes was displayed by ZZY-1 (Figure 8), more so than GD-6, which explains the reduction in phosphorus concentration in the plants exposed to As toxicity. Arsenic utilizes phosphate transporters to enter the plant’s system, for that reason, less As accumulating genotype (i.e., ZZY-1), tended to adopt an avoidance strategy by reducing the expression levels of such genes (Kumar et al., 2011; Ghorbani et al., 2021), even though its development was interfered considerably but the plant still managed to survive and grow slowly, which was the absolute reverse effect observed for GD-6 genotype. Contrariwise, *Serendipita indica*-mediated burgeoning of ZZY-1 and GD-6 plants was predicated on an increased uptake of macro and micro nutrient, particularly P, very much compatible with the upregulation of phosphate transporters in roots such as PHT1-1 and PHT1-5, as described previously for *Arabidopsis* and rice (Johnson et al., 2014). Bajaj et al. (2018) declared a significant increase in the nitrogen, phosphorus and potassium concentration in aboveground tissues of *S. indica*-colonized soybean plants, along with some other vital nutrients, such as manganese, zinc, calcium, magnesium, and especially iron. It has also been confirmed that in symbiosis with roots, *S. indica* acts as a shield or immobilize/sequesters HMs in its vacuoles, and our results regarding As translocation factor (Figure 3) correspond well with those of previous studies (Mohd et al., 2017). By immobilizing As in the roots, *S. indica* prevents its translocation to the aerial parts and subsequent dysfunction of photosynthetic organs, which might, otherwise, lead to reduced chlorophyll biosynthesis, distorted chloroplast membranes and reduced photosynthetic rate (Gusman et al., 2013). Improvement of *Gs* by *S. indica* has also been documented in different plants under various stresses (Saddique et al., 2018; Bertolazi et al., 2019). During its colonization (at early symbiotic stages), extracellular adenosine 5′-triphosphate (eATP) accrues in the apoplast, which is known to promote stomatal opening and helps regulate plant’s growth and development along with the biotic and abiotic stress responses (Nizam et al., 2019).

Nam et al. (2021) provided evidence regarding Fe deficiency induced ROS generation as well as chlorosis that becomes more pronounced in the presence of phosphorus rather than its absence, which explains the improved photosynthetic capability and overall growth of plants in this study under *S.i* + As + P treatment as As hinders the uptake of Fe (Mousavi et al., 2020); neither the availability of P alone compensate for Fe deficit, nor does it represent a guarantee of counterbalancing As toxicity (Begum et al., 2008; Abbasi et al., 2021). Availability of P is, tacitly assumed to be, crucial for the concentrations of elements that are imperative for metabolic processes associated with photosynthesis, biomass growth and yield formation, such as Mg (Weih et al., 2021); plants colonized by *S. indica* combined with As and P benefitted much more than the ones without its symbiosis (i.e., As + P), not only in terms of nutrient uptake but also in the element concentration of Mg, which is indispensable for the formation of chlorophyll molecule (Weih et al., 2021). From this perspective, the augmented P proved rather inefficacious, when accompanied by As, for the studied genotypes. The ravaging effects of As on the physiological growth of plants has been delineated extensively in literature (Sharma et al., 2017; Li et al., 2018; Mousavi et al., 2020). Generally, the consensus is that As significantly reduces plant development and root/shoot biomass (Ghorbani et al., 2021). A parallel trend was observed in current study (Table 2; Figure 2), which validates the phytotoxicity of As on rice plants. Arsenic (AsV) vies with PO_4_^3−^ in ATP synthesis and replaces it, forming an unstable adenosine diphosphate-As (V), causing disruptions in cellular energy flow (Ye et al., 2017).

Even though plant inoculation with this endophytic symbiont results in phyto-promotion, the inoculation itself exerts a moderate biotic stress that activates defense mechanisms and induces systemic resistance (ISR) in plants (Gill et al., 2016; Shrivastava et al., 2018; Xu et al., 2018). Previous studies explain *S. indica*-mediated root protection through the stimulation of antioxidant system under stress, which likewise prompts developmental reprograming in response to the redox state of the roots and environmental cues (Vadassery et al., 2009; Harrach et al., 2013; Bakshi et al., 2017). It initially colonizes living cells *via* invagination of the cell-membrane, keeping the organelles intact but this biotrophic phase soon progresses into a cell-death related colonization phase (Qiang et al., 2012), rendering dominance of this endophyte over plant’s root (Sagonda et al., 2021; Ulhassan et al., 2022). Consequently, a characteristic corollary of *S. indica* symbiosis is an improved plant water homeostasis through an improved root system induction and access of mycelium to water, otherwise outside the reach of the root system, which contributes to higher A_max_ and gaseous exchange (Porcel et al., 2015). The present study results confirmed once again the effectiveness of *S. indica* in alleviating HM toxicity in rice plants concurring with numerous previous studies (Gill et al., 2016; Mohd et al., 2017; Sagonda et al., 2021). Ghorbani et al. (2021) proved in his study that *S. indica* can endow resistance against As toxicity to its host plant by a numbers of mechanisms, a very few of which have been understood by scientists, and among these, is the stimulation of enzymic activities within the plant cells and therefore regulation of stress by the plant natural defense mechanism; the same trends have been recorded in this study apropos of the stimulation of CAT, POD, and SOD in plants colonized by *S. indica*. In addition, other genes of interest, across the treatments and between genotypes, such as *OsMn-SOD*, *OsFe-SOD*, and *OsCu-ZnSOD* were also upregulated between genotypes and across treatments, indicating the relative induction of their activities and highly correlated with the elemental concentrations and the enzymic analysis witnessed across the treatments. The results gave us a new prospective, as we have noticed that the combined effects of the treatments perhaps can be more promising in the As-sensitive genotypes. Heavy metal ATPases, i.e., HMA2 and HMA3 partake in xylem transportation and vacuolar sequestration of HMs, respectively (Adil et al., 2020a). However, their association is not limited to Cd and Zn translocation only as the involvement, of HMA3 in particular, reportedly encompasses stress response, senescence as well as Fe-deficiency (Zeshan et al., 2021). Current study also showed upregulation in the expressions of both HMAs, especially under treatments with As, which validates the role of these genes in metal homeostasis.

Results from the ROS determination (Figure 6) gave us new perspectives to understand and explain the differential responses of the two rice genotypes and corroborate previous studies done by Karuppanapandian et al. (2011), Ghorbani et al. (2021) and Mohd et al. (2017), all supporting the fact that in stressful conditions, ROS production increases significantly (*p* < 0.05) and this consequently leads to systematic observation of poor growth parameters and bad performances of the plant. Symbiosis of *S. indica* does not induce oxidative burst rather represses it by triggering different enzymes that scavenge ROS (Vadassery et al., 2009). The acquired oxidative burst data correlated with growth parameters and ultrastructure images. The electron imaging of both genotypes tissues cells, revealed the important damaging effects caused by the different treatments on different scales, it was however, noted that the combined treatment effect was greater on root and shoot cellular organelles of GD-6 genotype as shown in Figure 3; these results correlate with the growth parameters discussed above. Taken together, data from this study implied the higher impact of treatments in GD-6 (As-sensitive genotype), surpassing ZZY-1 (As-tolerant genotype). The complex root-fungi cellular interactions necessitate constant recognition/signal exchange (Bonfante and Requena, 2011). Consistent with the fact that *S. indica* is a root colonizing endophyte, its cell wall extracts have been previously reported to induce sequential cytoplasmic and nuclear Ca^2+^elevations, preferably in the roots and marginally in the shoots (Vadassery et al., 2009). Inferentially, Ca^2+^ ions function as a second messenger in plant-signaling pathways to create a link between extracellular stimuli and intracellular responses (Adil et al., 2020b), thereby facilitating an improved plant performance. A comparatively higher concentration of calcium in both shoots and roots of *S. indica* inoculated plants with or without As stress imply toward the remarkable tendency of this fungus in forearming the plants to the anticipated agricultural challenges.

## Conclusion

In current study, the augmented provision of P assisted the susceptible genotype much more than the tolerant one. Moreover, the *S. indica* colonization without additional P was sufficient enough to support growth and photosynthetic aspects of less As accumulating genotype (ZZY-1). The *S. indica* symbiosis serves as a lynchpin by enabling plants to take up and utilize the available resources to their fullest and contributing to an increased P nutrition, improved plant growth and amelioration of As toxicity. High-P contents facilitated by the association of this endophyte convey enough energy to plants for their survival and produce enough biomass to produce a dilution effect. Albeit, for an in-depth assessment of the regulatory pathways that underlie the symbiotic association of *S. indica* with P amended arsenic stressed rice plants, further studies encompassing proteomic or metabolic approaches are imperative. The evidence gathered here portrays the significance of *S*. *indica* combined with P application as a model approach for integration in sustainable agriculture and the improvement of crop productivity in farmlands polluted with arsenic.

## Data availability statement

The original contributions presented in the study are included in the article/supplementary material, further inquiries can be directed to the corresponding author.

## Author contributions

SS and MFA: Experimental work, Methodology, Data analysis and Writing—original draft. QF and ZI: Methodology, Formal analyses and Software. FSS, DMB, NU and YO: Writing - review & editing. YG: Conceptualization, Funding acquisition and Writing—review & editing and IHS: Conceptualization, Supervision, Funding acquisition, Correspondence and Writing—review & editing. All authors have read and agreed to the published version of the manuscript.

## Funding

This research work was financially supported by the Sino-Pakistan Project NSFC (grant no. 31961143008), National Natural Science Foundation of China, International (Regional) Cooperation and Exchange Program, Research fund for International young scientists (grant no. 31750110462), Jiangsu Collaborative Innovation Center for Modern Crop Production (JCIC-MCP), China and Guizhou Provincial Academician Workstation of Microbiology and Health, Project Number: Guizhou Kehe Platform Talents[2020]4004.

## Conflict of interest

The authors declare that the research was conducted in the absence of any commercial or financial relationships that could be construed as a potential conflict of interest.

## Publisher’s note

All claims expressed in this article are solely those of the authors and do not necessarily represent those of their affiliated organizations, or those of the publisher, the editors and the reviewers. Any product that may be evaluated in this article, or claim that may be made by its manufacturer, is not guaranteed or endorsed by the publisher.
